# Drug Delivery and Visual Monitoring of Nd(ATA)-GelMA Composite Hydrogels

**DOI:** 10.3390/gels12070635

**Published:** 2026-07-16

**Authors:** Tongyu Qiu, Fengyuan Bian, Tong Meng, Wei Zhou, Weijie Zhang, Ming Ma, Yihu Wang, Bing Zhang

**Affiliations:** 1 Nano- and Micro-Functional Materials Research Laboratory, School of Light Industry Science and Engineering, Beijing Technology and Business University, Beijing 102488, China; twody51946@163.com (T.Q.); b110320@126.com (F.B.); m1598917188@163.com (T.M.); 2 Technical Institute of Physics and Chemistry, Chinese Academy of Sciences, Beijing 100190, China; zhangweijie@mail.ipc.ac.cn (W.Z.); maming@mail.ipc.ac.cn (M.M.); wyh8632@mail.ipc.ac.cn (Y.W.)

**Keywords:** Ln-MOFs, drug-loaded hydrogel, drug release, in vitro monitoring

## Abstract

In this study, taking NdCl_3_ and 2-amino-1,4-benzenedicarboxylic acid (H_2_ATA) as raw materials, a novel lanthanide metal–organic framework, Nd(ATA), was synthesized by the coprecipitation method. After loading antibiotic levofloxacin (LEV), Nd(ATA) was combined with GelMA hydrogel to prepare a drug-loaded composite hydrogel, LEV@Nd(ATA)-Gel, which can emit near-infrared fluorescence under excitation at 808 nm and possesses improved mechanical properties compared to pure GelMA hydrogel. LEV@Nd(ATA)-Gel exhibited high bactericidal activity and low cytotoxicity, with cell viability increased by 35% compared to the control group. The release rate of the loaded LEV was found increasing with the pH decreasing from 7 to 3, and demonstrated a potential responsiveness to wound microenvironment. Furthermore, drug delivery studies revealed a significant correlation with the fluorescence intensity of the composite hydrogel and the drug release behavior, and the extent of drug release was quantitatively captured by an in vitro imaging technology. This study successfully integrated the drug release with fluorescent signal of carrier, providing a highly sensitive and visualizable strategy for the development of internal wound adhesive.

## 1. Introduction

Medical gauze and bandages are easy to use and cost-effective; however, several deficiencies, including a high risk of infection, a lack of effectiveness on chronic wounds, and significant pain during dressing changes, have been limiting the sustainable usage of these traditional wound dressings [1]. With the rapid advancement of materials science, porous sponges, breathable films, and microneedle patches have successively emerged [2,3,4]. Due to rapid hemostasis, bacterial inhibition, and promotion of healing, these novel materials have become promising candidates for upgrading wound dressings. Therein, hydrogels are regarded as highly competitive alternatives for extracellular matrix-like microenvironments, with exceptional moisture-retaining capacity, excellent biocompatibility, and tunability [5].

As raw materials of hydrogels, sodium alginate, hyaluronic acid (HA), gelatin and so on typically exhibit drawbacks such as slow gelation, poor toughness and adhesion [6], which hardly meet the requirements for stable wound protection and long-term repair of chronic wounds [7]. By modifying the molecular structure, the cross-linking sites and modes of the raw materials were adjusted, and as a result the performance of hydrogels were effectively improved. Zhang et al. introduced dynamic aconitic acid bonds, disulfide bonds, and maleic acid groups into sodium hyaluronate, resulting in a hydrogel with a dual-network structure that combines rapid gelation with dermis-like mechanical strength [8]. Gelatin-based hydrogels treated with methacrylation rapidly solidified under UV light, significantly enhancing the hydrogel’s mechanical strength, structural stability, and resistance to swelling [9]. The introduction of phenolic hydroxyl groups onto polycarboxylic amino-based substrates such as HA and sodium alginate allows for the formation of hydrogen bonds, hydrophobic interactions, and π-π stacking with surface groups on tissues, thereby significantly optimizing wet adhesion [10,11]. The modified hydrogels demonstrated significant improvements in mechanical properties and adhesion, exhibiting multifunctional capabilities such as rapid hemostasis, immunomodulation, and use as tissue engineering scaffolds.

At the same time, the antimicrobial activity as a key property of hydrogels is also attracting significant attention [12]. Currently, the most common approach involves directly loading antimicrobial agents such as antibiotics [13] and curcumin [14] into hydrogels; however, this method suffers from drawbacks such as severe burst release and insufficient targeting, and cannot satisfy the long-term, highly effective antimicrobial protection in chronically infected wounds [15]. By grafting functional groups such as phenylboronic acid onto the matrix materials, controllable drug-releasing behavior of the hydrogel responding to glucose, Reactive Oxygen Species (ROS), and pH has been achieved [16]. Furthermore, by loading drugs into functional nanomaterials such as graphene [17] and metal–organic frameworks (MOFs) [18], and then combining with hydrogels to construct a two-stage drug delivery system, drug burst release has been more effectively suppressed, and drug-responsive release based on internal/external stimuli enhances practical value [19]. The release behavior of loaded antimicrobial drugs plays a decisive role in wound healing effect, and studies on drug release kinetics can provide important guidance for the customization of drug-loaded hydrogels and the regulation of exogenous stimuli [20].

Traditional in vitro studies of hydrogel drug release behavior involve periodically collecting Phosphate-Buffered Saline (PBS) extraction solutions and then measuring drug concentrations by liquid-phase analysis, UV–visible spectrophotometry, or fluorescence bleaching-recovery assays [21,22,23]. However, in vitro conditions struggle to effectively simulate the numerous additional variables and influencing factors present in the in vivo environment, and thus cannot accurately reflect in situ drug release behavior inside the organism. Current in vivo drug delivery monitoring techniques generally employ conjugation of the drug with markers with special optical or magnetic properties, such as carbon dots, gold nanoclusters, or gadolinium, and utilize fluorescence or nuclear magnetic resonance equipment to measure the in vivo levels of markers to reflect drug release [24,25,26]. However, the currently reported markers still have some shortcomings in terms of the drug delivery process, tissue penetration and response sensitivity [27,28,29], and the development of new marker materials is an urgent task. Due to their high adsorption capacity and excellent fluorescent properties, rare-earth metal–organic frameworks (Ln-MOFs) have been widely applied in fields such as environmental monitoring, food safety, and tissue imaging [30,31,32]. Lanthanide metals can form strong coordination interactions with drug molecules, enabling the drug molecules not only to be passively adsorbed onto the pores but also to be stably anchored to the framework via chemical bonds, thereby significantly improving drug loading efficiency. Incorporating Ln-MOFs into the construction of drug-loaded hydrogels is bound to provide design strategies for wound adhesives that combine smart drug delivery with in situ monitoring.

**Figure 1:**
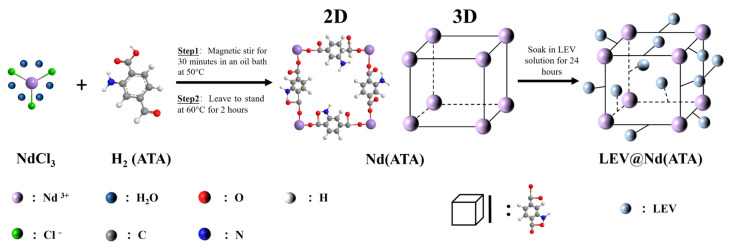
LEV@Nd(ATA) Composite Image.

Therefore, this study selected neodymium chloride (NdCl_3_) and 2-amino-1,4-benzenedicarboxylic acid (H_2_ATA) as raw materials to synthesize the rare-earth metal–organic framework Nd(ATA) via the coprecipitation method (Figure 1). LEV was loaded into Nd(ATA) through physical adsorption, and the responsiveness of drug release behavior to environmental pH was determined. Subsequently, the drug-loaded Nd(ATA) was combined with GelMA hydrogel to develop a wound adhesive equipped with controlled drug release and in situ imaging. Meanwhile, the adhesion, biocompatibility, and antibacterial activity of the composite hydrogel were researched. Finally, based on the photoinduced electron transfer or intramolecular filtration effects between the antibiotic molecules and the Ln-MOFs, the correlation between drug release behavior and fluorescence properties was regulated, thereby enabling the visualization of drug release behavior in vivo.

## 2. Results and Discussion

### 2.1. Characterization of Nd(ATA) and GelMA

**Figure 2:**
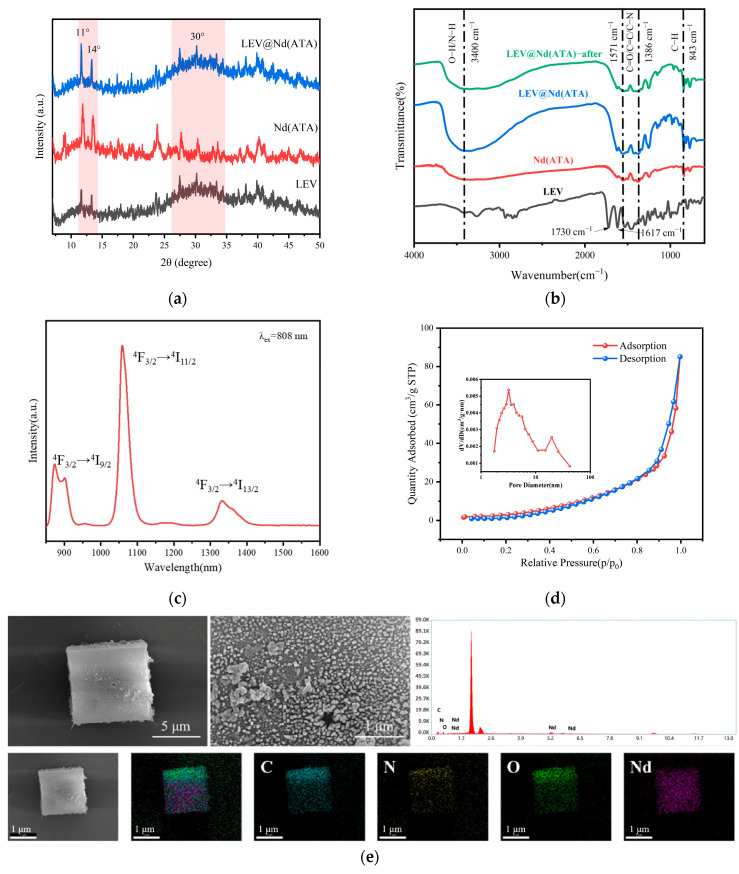
Characterization of physicochemical properties: (**a**) XRD of LEV, Nd(ATA) and LEV@Nd(ATA); (**b**) FTIR spectrum of LEV, Nd(ATA), LEV@Nd(ATA) and LEV@Nd(ATA) after drug release; fluorescence emission spectrum (**c**), BET adsorption (**d**), and SEM image (**e**) of Nd(ATA).

X-ray diffraction analysis indicates (Figure 2a) that the coprecipitated product exhibited characteristic diffraction peaks of Nd(ATA) at 2θ = 9.68°, 12.29° and 14.25°, and we calculated the size of the Nd(ATA) polycrystalline material using the Debye–Scherrer equation [33] to be D = 28.3 nm. As reported in the literature, the synthesized Ln-MOFs exhibit good crystallinity [34]. In the infrared spectrum of Nd(ATA), a broad peak appears in the 3500–3000 cm^−1^ region, corresponding to the O-H stretching vibration of the carboxylic acid and the N-H stretching vibration of the primary amine groups on the ligand benzene rings (Figure 2b). Upon excitation with 808 nm light, the fluorescence spectrum of Nd(ATA) exhibited three main characteristic emission peaks of Nd^3+^ (Figure 2c), located at 873 nm (4F_3/2_ → 4I_9/2_), 1058 nm (4F_3/2_ → 4I_11/2_) and 1332 nm (4F_3/2_ → 4I1_3/2_) [35] indicating near-infrared emission properties. The N_2_ adsorption–desorption isotherm of Nd(ATA) exhibits a Type IV isotherm profile (Figure 2d), indicating that the material is mesoporous. Its BET specific surface area is 13.51 m^2^/g, with an average adsorption pore diameter of approximately 36.73 nm (inset in Figure 2d), suggesting that the material possesses a certain adsorption capacity. The SEM image shows that Nd(ATA) exhibits a cubic morphology with a particle size of approximately 5 μm (Figure 2e); under high magnification (1 μm), numerous depressions and pores can be observed on its surface. Elemental analysis indicates that Nd, C, N and O are uniformly distributed within Nd(ATA).

In contrast, the XRD pattern of LEV@Nd(ATA) resembles a physical superposition of LEV and Nd(ATA), perfectly confirming the success of the complex formation (Figure 2a). It retains all the sharp diffraction peaks characteristic of Nd(ATA), indicating that the original crystal structure of Nd(ATA) was not disrupted during the incorporation of the drug, thus maintaining excellent structural stability. At the same time, it exhibits a ‘bun-shaped’peak in the 26–35° range, identical to that of LEV. Overall, the spectrum of the LEV@Nd(ATA) complex exhibits both the characteristic crystalline peaks of Nd(ATA) and the superimposed broad amorphous background of LEV. The coexistence and superposition of these characteristic peaks confirm that LEV has been successfully loaded onto Nd(ATA) and that the main structure of Nd(ATA) remains stable after loading.

Infrared spectroscopy LEV@Nd(ATA) further confirms that LEV has been successfully loaded onto Nd(ATA) (Figure 2b). The 3600–3300 cm^−1^ region corresponds to O-H/N-H stretching vibrations. In LEV@Nd(ATA), the absorption peaks in this region are noticeably broader and more intense, indicating that the addition of LEV has introduced more O-H/N-H-related vibrations and may have formed hydrogen bonds. These changes in this region serve as key evidence for the interaction between LEV and Nd(ATA). The 1700–1350 cm^−1^ region likely involves carboxyl C=O stretching vibrations, aromatic ring C=C skeletal vibrations, and related C-N vibrations. As shown in the figure, the peak shapes in LEV@Nd(ATA) are generally similar to those in Nd(ATA), but the increased peak intensity may be due to the fact that the carboxylate framework of Nd(ATA) remains intact, and the LEV carbonyl group may interact with Nd^3+^, -NH_2_, or surface sites in Nd(ATA). The region between 900 and 700 cm^−1^ corresponds to the aromatic ring C-H bending vibration. Distinct characteristic absorption peaks are observed for both LEV and Nd(ATA) in this region, and LEV@Nd(ATA) retains similar absorption signals with enhanced intensity, indicating that the aromatic heterocyclic structure or characteristic fingerprint peaks of LEV have appeared in the composite material. This FTIR spectrum supports the successful loading of LEV onto Nd(ATA), as the spectrum of LEV@Nd(ATA) retains both the skeletal absorption peaks of Nd(ATA) and the characteristic absorption peaks of LEV. Additionally, some peak positions have shifted and peak shapes have broadened, suggesting that hydrogen bonding, coordination, or intermolecular interactions may exist between the two, rather than simple mechanical stacking.

**Figure 3:**
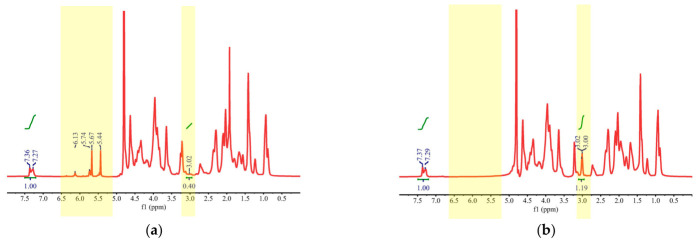
^1^H NMR spectra of gelatin and GelMA: (**a**) gelatin, (**b**) GelMA.

MA confers cross-linkable properties on gelatin whilst preserving its biological activity, such as the RGD sequence, thereby endowing GelMA with excellent biocompatibility. A comparative analysis of the ^1^H NMR spectra of gelatin and GelMA (Figure 3a,b) revealed the appearance of an MA alkenyl proton peak at 5.5–6.5 ppm in GelMA, alongside a marked decrease in the intensity of the lysine residue peak at 2.9–3.1 ppm. This indicates that the amino groups have been consumed to form ester bonds, confirming the successful synthesis of GelMA. By calculating the change in the integral value at 2.9–3.1 ppm using the constant phenylalanine peak at approximately 7.3 ppm, the substitution degree of GelMA was determined to be approximately 66%.

### 2.2. Mechanical Properties of the Composite Hydrogel

**Figure 4:**
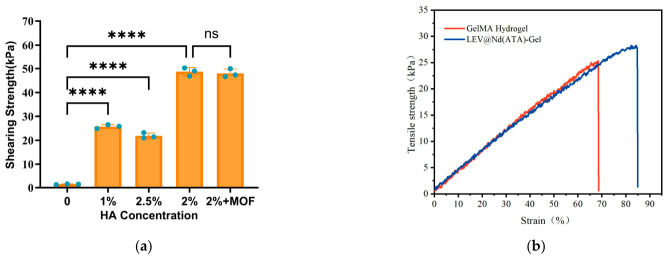
(**a**) The effect of hyaluronic acid concentrations of 0%, 1%, 2%, and 2.5% on the adhesion properties of composite hydrogels, as well as the effect of adding MOFs to a 2% hyaluronic acid solution; (**b**) tensile strength testing of GelMA hydrogels. Data are expressed as mean ± SD (n = 3; ns: *p* > 0.05; ****, *p* ≤ 0.0001).

The adhesive properties of the hydrogel arise from several factors. The primary mechanism is the bioadhesion of high-molecular-weight HA to tissue [36,37]. Secondly, the carboxyl groups in the hydrogel can bind to tissue via electrostatic interactions [38]. Furthermore, hydrogen bonding between the functional groups in the hydrogel and the tissue also enhances the hydrogel’s adhesion [39,40]. Figure 4a shows that the strength of the hydrogel without added HA was only 1.42 kPa; when the HA content was increased from 1% to 2%, the hydrogel’s adhesion increased to 50 kPa. However, as the HA content was further increased to 2.5%, the hydrogel’s adhesion began to decline. This may be due to the local aggregation of high-molecular-weight HA, which fails to disperse uniformly throughout the system and is thus unable to form an effective interactive network with GelMA, resulting in reduced adhesion. The incorporation of LEV@Nd(ATA) into a hydrogel containing 2% HA largely preserved the hydrogel’s original viscosity, indicating that its spatial structure remained intact. The maximum adhesion performance measured under experimental conditions was 45 kPa, approximately three times that of commercial fibrin glue [36].

Compared with pure hydrogels, the tensile strength of the composite hydrogel increased by approximately 12%, reaching around 28 kPa (Figure 4b), which is roughly three times that of commercial fibrin glue [41]. The increase in strength may be attributed to the fact that MOFs promote the formation of ordered secondary structures in GelMA, significantly increasing β-sheet structures and reducing the presence of random coiled structures [42,43]. Furthermore, the presence of MOFs as fillers results in a denser cross-linked network. The synergistic interaction between components at the protein secondary structure level, combined with the increased network density, contributes to the higher mechanical strength of the composite hydrogel. Alongside the increase in tensile strength, the hydrogel’s toughness has also improved to some extent, as evidenced by a slightly longer elongation at break compared to the non-composite hydrogel.

### 2.3. Biocompatibility of the Composite Hydrogel

**Figure 5:**
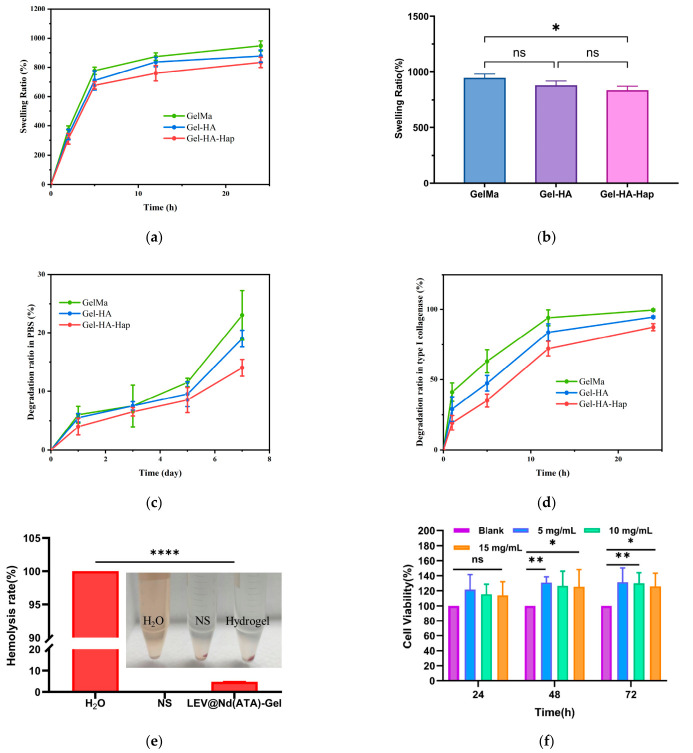

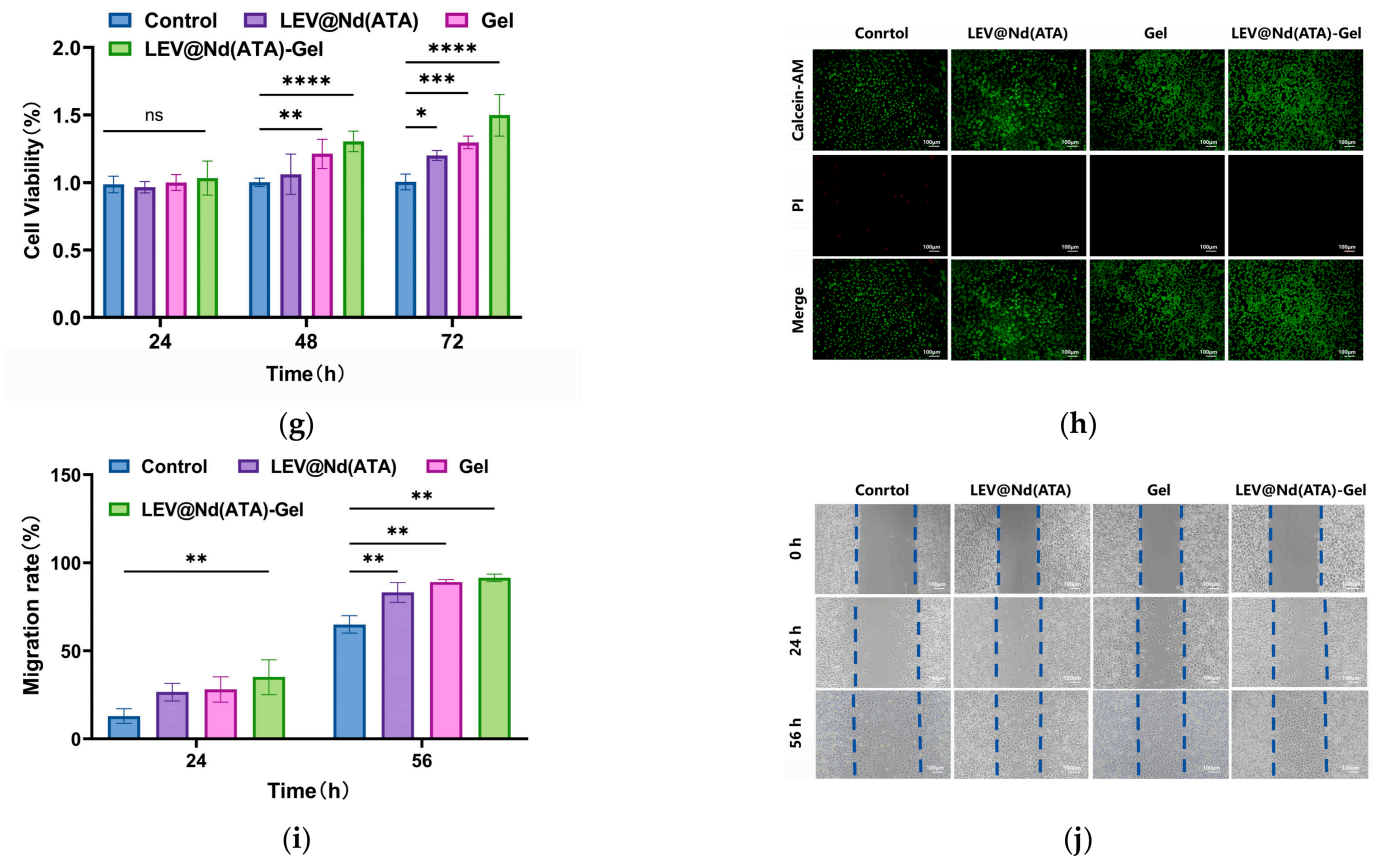
Swelling curves (**a**), swelling rates (**b**), and degradation profiles of GelMa, Gel-HA (HA concentration: 2%), and Gel-HA-Hap (HA concentration: 2%, Hap concentration: 5%) in PBS over 24 h, as well as their degradation profiles in PBS (**c**) and PBS containing 2 U/mL Type I collagenase (**d**). Biocompatibility testing of LEV@Nd(ATA): (**e**) cytotoxicity and (**f**) hemolysis; cell viability (**g**), live/dead staining (**h**), cell migration rate (**i**), and migration images (**j**) of L929 cells treated with control, LEV@Nd(ATA), Gel(no LEV@Nd(ATA)) and LEV@Nd(ATA)-Gel. Data are expressed as mean ± SD (n = 3; ns: *p* > 0.05; *, *p* < 0.05; **, *p* < 0.01; ***, *p* ≤ 0.001; ****, *p* ≤ 0.0001).

In wound dressings, hydrogels require high swelling capacity to absorb, exudate, and maintain a moist wound environment [44]. As shown in Figure 5a, the mass of the hydrogel increases rapidly after immersion in water, and the swelling ratio essentially stabilizes within 24 h. The maximum swelling rates for GelMa, Gel-HA, and Gel-HA-Hap were 946.6 ± 34.5%, 876.6 ± 47.1%, and 833.3 ± 35.1%, respectively (Figure 5b). Low-concentration hydrogels exhibit higher equilibrium swelling rates, which may be attributed to their larger pore sizes and looser network structures, allowing them to absorb more water.

To assess the stability of the hydrogels in vivo, we tested their degradation properties. When immersed in PBS (Figure 5c), all three hydrogels degraded at a relatively slow rate and retained a relatively intact structure for one week. Since the pure GelMa group contained no structural reinforcing agents, its degradation rate was faster than that of the other two groups, at 24 ± 4.2%. In contrast, the Gel-HA-Hap group exhibited the highest strength among the three samples, resulting in a slower degradation rate of only 14 ± 1.4%. When immersed in a PBS solution containing Type I collagenase, the hydrogels underwent rapid degradation (Figure 5d). At 1 h, the degradation rates for GelMa, Gel-HA, and Gel-HA-Hap were 41 ± 6.5%, 29 ± 8.5%, and 19.3 ± 5.1%, respectively. After 24 h, the degradation rates for each group were 99.5 ± 0.7% (GelMa), 94.5 ± 0.7% (Gel-HA), and 87.3 ± 2.5% (Gel-HA-Hap).

Figure 5e illustrates the haemolysis of New Zealand rabbit erythrocytes by the composite hydrogel. As shown in the inset, the supernatant in both the saline negative control group and the composite hydrogel group exhibited only a slight reddish tinge, with a deposit of blood cells visible at the bottom of the centrifuge tubes, indicating minimal haemolysis. In contrast, the supernatant of the deionised water positive control group was red, and the red blood cells at the bottom had disappeared, indicating complete haemolysis. A comparative analysis of haemolysis rates revealed that the haemolysis rate in the composite hydrogel group was only 4.76%, a significant difference from the deionised water group. This value is below the 5% safety threshold [45], thereby meeting the basic biosafety standards for biomedical applications.

Good biocompatibility is crucial for the medical application of hydrogel wound adhesives in the human body [46]. Figure 5f shows the relative cell viability of L929 cells after 24, 48 and 72 h of culture in extract solutions of the composite hydrogel at different concentrations. None of the three concentrations of extract solution exhibited significant cytotoxicity towards the cells; on the contrary, a proliferative effect was observed. Furthermore, as the culture time increased, cell viability rose from 20% to 35% compared to the control group. Gelatin is derived from collagen and possesses extremely high biocompatibility [47]. GelMA enhances the mechanical properties of gelatin after gelation whilst retaining its excellent biocompatibility; simultaneously, as a bioactive substrate for matrix metalloproteinases, GelMA promotes cellular remodeling, migration, differentiation and proliferation [48]. Furthermore, hyaluronic acid in the hydrogel formulation is a major component of the extracellular matrix. This non-sulphated glycosaminoglycan plays a key role in processes such as cell proliferation, growth, survival, polarization and differentiation [49]. The two components act synergistically to promote fibroblast proliferation.

CCK-8 was used to measure the relative cell viability of L929 cells after 24, 48, and 72 h of culture in LEV@Nd(ATA), Gel(no LEV@Nd(ATA))and LEV@Nd(ATA)-Gel extracts (Figure 5g). The relative cell viability of all samples remained above 100% throughout the 72 h period, demonstrating the absence of cytotoxicity. The morphology of L929 cells after 24 h of culture in the gel extract was observed via live/dead staining, and the results of the live/dead staining further confirmed (Figure 5h) that after three days of culture, all groups showed predominantly green live cells with a small number of red dead cells, indicating that the material exhibits good cellular compatibility. The results of the CCK-8 assay and live/dead cell staining showed that the cell viability in the LEV@Nd(ATA)-Gel group was significantly higher than that in the control group, and the live cell density was also significantly increased.

The results of the analysis of cell migration in L929 cells after 56 h in the extract solutions of LEV@Nd(ATA), Gel and LEV@Nd(ATA)-Gel are shown in Figure 5i. The image is shown in Figure 5j, with the control group being DMEM cell culture medium. The results indicate that the area of the cell scratch decreased over time. The average cell migration rate of LEV@Nd(ATA)-Gel was higher than that of the control group at 56 h, indicating that LEV@Nd(ATA)-Gel enhances cell migration capacity and has the potential to accelerate wound healing.

### 2.4. Drug Sustained Release

#### 2.4.1. In Vitro Release Behavior

**Figure 6:**
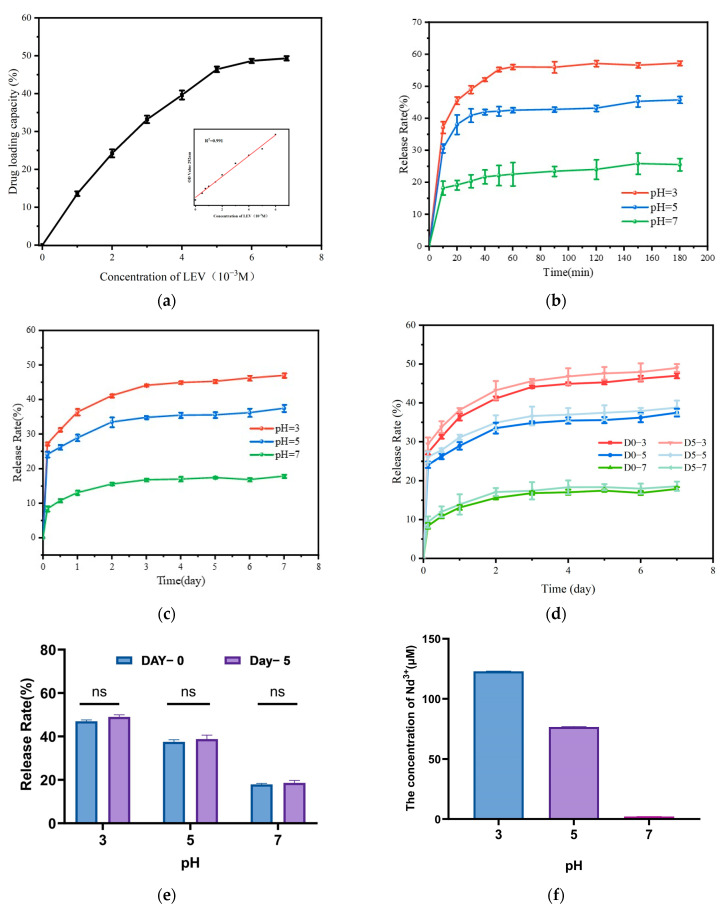
(**a**) Drug loading capacity of Nd(ATA) at different LEV concentrations, inset: calibration curve for LEV concentration; plots showing drug release from (**b**) LEV@Nd(ATA) and (**c**) LEV@Nd(ATA)-Gel at pH = 3, 5, 7 levels; drug release curves (**d**) and drug release rates (**e**) of LEV@Nd(ATA)-Gel after 0 days (D0) and 5 days (D5) at pH 3, pH 5, and pH 7; (**f**) ICP-OES: concentration of Nd^3+^ released into the supernatant of LEV@Nd(ATA) after 48 h in solutions of different pH values. Data are expressed as mean ± SD (n = 3; ns: *p* > 0.05).

The normal pH of the human body is around 7.4, but there are significant variations in pH across specific organs (stomach: 1.5–3.5; small intestine: 4.5–6.5; large intestine: 6.0–8.0). Consequently, the drug release behavior of the drug-loaded hydrogel under different pH conditions directly determines the efficacy of wound healing. At the same time, based on the drug loading formula, the final drug loading of Nd(ATA) was 46.7% (Figure 6a), which is higher than that of other MOF drug delivery systems [50]. Figure 6b,c illustrate the release profiles of LEV under two loading modes in different pH environments. Overall, as the pH decreases from 7 to 3, both loading modes of LEV exhibit accelerated release. This is primarily due to the ATA ligand in Nd(ATA) containing carboxyl groups (pKa1 ≈ 3, pKa2 ≈ 5) and amino groups (pKa ≈ 9–10). At low pH (e.g., pH = 3), partial protonation of the carboxyl groups leads to the breaking of the coordination bonds in the organic framework and structural collapse, resulting in rapid LEV release. At neutral pH (e.g., pH = 7), the carboxyl groups are fully deprotonated, the framework structure is more stable, and drug release is slowed.

However, the drug release from LEV@Nd(ATA) in PBS solutions at pH 3, 5 and 7 reached equilibrium at around 60 min (Figure 6b), and the release amounts are 56%, 45%, and 25% respectively, whereas for LEV@Nd(ATA)-Gel (Figure 6c), the drug release over 3 days was approximately 46%, 37% and 16%, respectively, reaching equilibrium at around 7 days. This difference can be attributed to the fact that LEV@Nd(ATA)-Gelemploys a two-stage loading mechanism, where the drug must first be released from the pores of the primary carrier (MOFs) before passing through the secondary carrier (hydrogel) to reach the wound site; consequently, both its release rate and equilibrium release rate are lower than those of LEV@Nd(ATA). Therefore, by tailoring the drug loading in the composite hydrogel to the specific pH conditions of different organs and the characteristics of the wound, it is possible to achieve more effective wound healing and antimicrobial effects.

After storing the LEV@Nd(ATA)-Gel at 4 °C for five days, drug release tests were conducted in environments with pH 3, pH 5, and pH 7. The results were compared with those of LEV@Nd(ATA)-Gel that had not been stored to verify the fluorescence stability of LEV@Nd(ATA)-Gel after prolonged storage. As shown in Figure 6d, the drug release rate of LEV@Nd(ATA)-Gel after five days of storage was slightly higher than that of LEV@Nd(ATA)-Gel that had not been stored; however, there was no significant difference between the two groups (Figure 6e).

Although this phenomenon intuitively reveals the structural instability of Nd(ATA) under extremely acidic conditions (pH = 3), the core focus of this study is on the physiologically relevant weakly acidic pH range (5–7). Under these conditions, the acidic environment primarily acts on the hydrogen-bond interactions between LEV and the carrier in LEV@Nd(ATA), thereby promoting controlled drug release [51]. At the same time, we used ICP-OES to quantitatively analyze the Nd^3+^ concentration in the supernatant after 48 h of LEV@Nd(ATA) release under acidic conditions; the Nd^3+^ concentrations released at pH 3, 5, and 7 were 122.8 μM, 76.6 μM, and 2.2 μM (Figure 6f), respectively. Compared to the Nd^3+^ content in 2 mg/mL of LEV@Nd(ATA), these concentrations can be considered trace amounts. According to the existing literature [52], the acute cytotoxicity threshold of neodymium chloride for RAW 264.7 macrophages is as high as 700 μM, indicating that the released neodymium ion concentrations (particularly 76.6 μM and 2.2 μM at pH = 5 and pH = 7, respectively) are far below the levels required to induce acute toxicity in normal cells. Furthermore, since LEV@Nd(ATA) is encapsulated in a hydrogel, trace amounts of free Nd^3+^ are trapped by the hydrogel and are not directly released into the human body. It should be noted that although the ICP-OES results indicate that the release of Nd^3+^ was at a low level under the experimental conditions of this study, this research is limited to a preliminary evaluation at the in vitro cellular level. Direct experimental data are currently lacking regarding the systemic distribution of released ions within the body, their tendency to accumulate in specific organs, long-term metabolic clearance pathways, and potential long-term toxic effects. Therefore, the aforementioned in vitro biocompatibility conclusions should be regarded as exploratory findings, and caution must be exercised when extrapolating them to in vivo applications. This limitation will be addressed in subsequent studies using animal models and long-term follow-up experiments.

#### 2.4.2. In Vitro Correlation of Fluorescence Intensity and Drug Release

**Figure 7:**
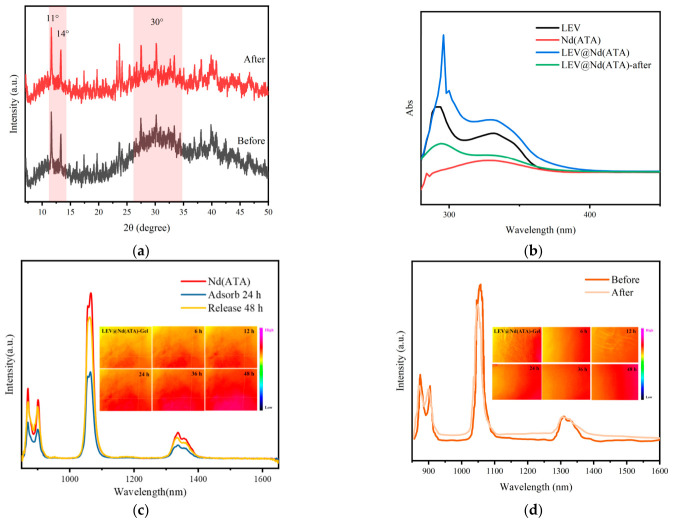
(**a**) XRD patterns of LEV@Nd(ATA) before and after drug release at pH 5. (**b**) UV spectra of LEV, Nd(ATA), LEV@Nd(ATA) and LEV@Nd(ATA) after drug release. (**c**) Fluorescence changes in Nd(ATA) before and after LEV adsorption (24 h) and release (48 h); the inset shows the near-infrared emission of LEV@Nd(ATA)-Gel at different drug release times, as captured by a CCD camera. (**d**) Comparing the changes in Nd(ATA) fluorescence before and after five days of storage, the figure shows the near-infrared emission spectra of LEV@Nd(ATA)-Gel after five days of storage, captured by a CCD camera at different drug release times.

Since LEV@Nd(ATA) contains neodymium ions, the degradation of Nd(ATA) in an acidic environment may lead to the release of neodymium, thereby causing potential toxicity issues. We subjected LEV@Nd(ATA) to a 24 h drug release experiment in a pH 5 environment. After drying, we compared the XRD patterns of LEV@Nd(ATA) before and after the experiment (Figure 7a). We found that only the intensity of the 30° peak, which is characteristic of LEV, had slightly decreased; the overall structure showed no significant changes, confirming that the overall structure of LEV@Nd(ATA) is highly stable.

As there is no overlap between the absorption spectrum of LEV and the fluorescence emission spectrum of Nd^3+^, factors such as fluorescence energy transfer and internal quenching can be ruled out. Furthermore, based on the UV spectra of LEV, Nd(ATA), and LEV@Nd(ATA) (Figure 7b), we observed a significant increase in absorbance, indicating that a chemical interaction occurred between LEV and Nd(ATA) in the ground state or that a ground-state complex was formed [53]. It is speculated that LEV was adsorbed into the pores of Nd(ATA) and interacts with active sites on Nd(ATA) via hydrogen bonding [54]. Based on the infrared spectrum shown in Figure 2b above, we had previously speculated that hydrogen bonding, coordination, or intermolecular interactions might exist between LEV and Nd(ATA), rather than the results being simply due to mechanical superposition. At the same time, we also found that after drug release, both the hydrogen-bond strength in the infrared spectrum and the peak intensity in the ultraviolet spectrum of LEV@Nd(ATA) decreased, demonstrating that the dissociation of LEV led to a reduction in the number of hydrogen bonds in LEV@Nd(ATA). This interaction alters the local chemical environment surrounding the Nd^3+^ ion, potentially providing a new vibrational relaxation pathway for the excited-state Nd^3+^, dissipating the excitation energy as thermal energy and thereby causing fluorescence quenching of Nd(ATA) [55].

The Nd^3+^ ions in Nd(ATA) are capable of emitting in the near-infrared II region under 808 nm excitation. The study found that the fluorescence intensity of the composite hydrogel decreased significantly after loading with LEV, but recovered to approximately its initial level 48 h after drug release (Figure 7c). Based on the relationship between drug release and the near-infrared fluorescence intensity of Nd(ATA), imaging studies of drug delivery using LEV@Nd(ATA)-Gel were conducted. As can be observed from the inset in Figure 7c, the initial composite hydrogel exhibits a relatively low-energy yellow color; as the drug is continuously released, the color of the image gradually shifts towards a high-energy red. When the release time reached 2 days, higher-energy purple hues were observed locally, indicating that the fluorescence intensity of Nd(ATA) had significantly increased due to the release of LEV.

At the same time, we found that the fluorescence of LEV@Nd(ATA)-Gel after five days of storage showed no significant difference from that observed previously (Figure 7d). Furthermore, as can be seen in the inset of Figure 7d—similar to the inset in Figure 7c—the initial composite hydrogel exhibited a relatively low-energy yellow color, which gradually shifted toward a high-energy red as the drug was continuously released. When the release time reached 2 days, higher-energy purple fluorescence was also observed locally, indicating that the Nd(ATA) fluorescence intensity did not diminish despite the prolonged storage of the LEV@Nd(ATA)-Gel.

### 2.5. In Vitro Antimicrobial Properties of Composite Hydrogel

**Figure 8:**
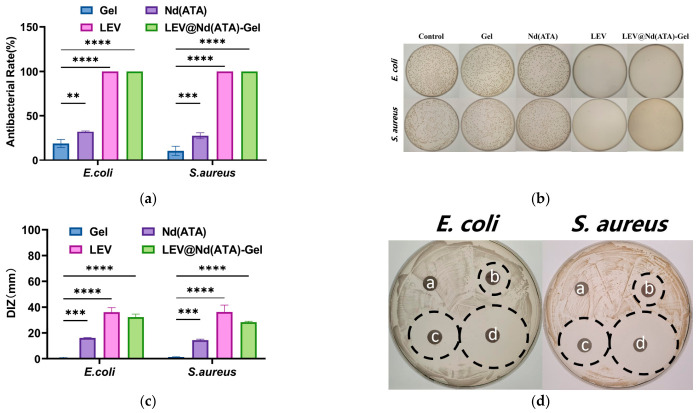

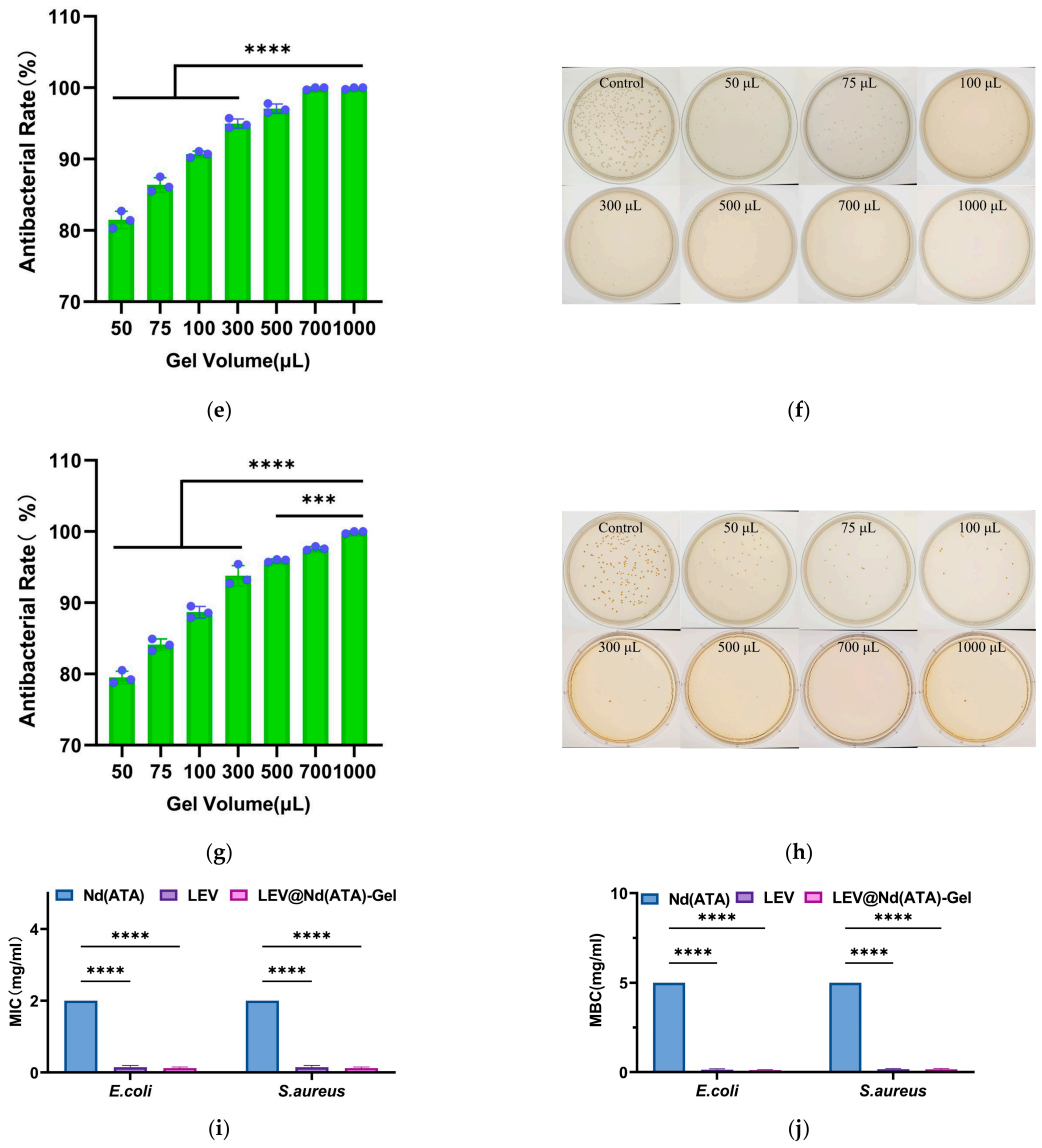
Antibacterial rates (**a**), antibacterial profiles (**b**), DIZ (**c**), and antibacterial activity (**d**) of Gel, Nd(ATA), LEV and LEV@Nd(ATA)-Gel against *E. coli* and *S. aureus*. Antibacterial rates (**e**) and antibacterial profiles (**f**) of LEV@Nd(ATA)-Gel at different concentrations against *E. coli*, and antibacterial rates (**g**) and antibacterial profiles (**h**) against *S. aureus*; MIC (**i**) and MBC (**j**) values for Nd(ATA), LEV, and LEV@Nd(ATA)-Gel. Data are expressed as mean ± SD (n = 3; **, *p* < 0.01; ***, *p* ≤ 0.001; ****, *p* ≤ 0.0001.).

Escherichia coli (*E. coli*) and Staphylococcus aureus (*S. aureus*) are two common bacteria encountered during the wound healing process [56]; infection with these bacteria can lead to inflammation and other adverse reactions in the human body. As shown in Figure 8a,b, the Gel group exhibited a relatively low antibacterial rate of only about 15%, while the Nd(ATA) group showed an antibacterial rate of approximately 30%, demonstrating weak antibacterial activity. In contrast, both the LEV and LEV@Nd(ATA) groups achieved an antibacterial rate of 100%, representing a highly significant difference compared to the pure gel group. This confirms that LEV@Nd(ATA)-Gel possesses excellent and broad-spectrum bactericidal capabilities; after being encapsulated and integrated into the gel, LEV perfectly retains its original high-efficiency antibacterial activity. The LEV@Nd(ATA)-Gel enables the loading and release of the antibiotic LEV; this study assessed the in vitro antimicrobial efficacy against these two bacteria.

The antibacterial activity of Gel, Nd(ATA), LEV, and LEV@Nd(ATA)-Gel was determined using the diameter of inhibition zone (DIZ) method. As shown in Figure 8c, there was no significant difference in the DIZ sizes produced by the four components against these two bacterial strains. The pure Gel group exhibited almost no DIZ, while Nd(ATA) demonstrated a slight antibacterial effect compared to the pure Gel group(Figure 8d). Since the concentration of LEV used in the DIZ test was 1 mg/mL—which is higher than the LEV content in LEV@Nd(ATA)-Gel—the DIZ of LEV was larger than that of LEV@Nd(ATA)-Gel. These DIZ results further confirm that LEV@Nd(ATA)-Gel possesses excellent, broad-spectrum bactericidal activity.

When the volume of the bacterial suspension was 1000 μL (Figure 8e,f), a gel volume of 700 μL was sufficient to achieve an inhibition rate of over 99% against *E. coli*, whereas a gel volume of 1000 μL was required to achieve an inhibition rate of over 99% against *S. aureus* (Figure 8g,h). This is because *E. coli* is a Gram-negative bacterium, whereas Staphylococcus aureus is a Gram-positive bacterium. As a fluoroquinolone antibiotic, LEV is more effective against E. coli than against Staphylococcus aureus; consequently, a smaller gel volume is required [57].

As shown in Figure 8i, the minimum inhibitory concentration (MIC) of Nd(ATA), LEV, and LEV@Nd(ATA)-Gel were 2000.00, 100, and 100 μg/mL, respectively. Based on the minimum bactericidal concentration (MBC) experimental results (Figure 8j), the MBC values for LEV and LEV@Nd(ATA)-Gel were consistent with their corresponding MIC values. However, since Nd(ATA) exhibits weaker antibacterial activity than LEV, the final MBC for Nd(ATA) was 5 mg/mL.

## 3. Conclusions

This study designed a multifunctional wound adhesive, LEV@Nd(ATA)-Gel, based on a composite hydrogel of the neodymium-ion organic framework Nd(ATA), which is intended for the treatment of bacterial infections in wounds whilst enabling real-time monitoring of drug release in vitro. The synthesized gelatin-modified GelMA material possesses excellent mechanical properties and adhesive strength, effectively protecting wound tissue. Nd(ATA), which emits in the near-infrared II region, was loaded with the antimicrobial agent LEV and incorporated into the hydrogel, endowing the LEV@Nd(ATA)-Gel with superior antimicrobial activity and biocompatibility, whilst enabling pH-responsive drug release dependent on the microenvironment. This demonstrates potential for antimicrobial treatment of wounds within the microenvironment. By utilizing the static quenching effect of the adsorbate LEV on Nd(ATA), we successfully achieved real-time in vitro monitoring of drug release via fluorescence imaging enhancement. In summary, this multifunctional composite hydrogel not only demonstrates significant capabilities in terms of mechanical properties, biocompatibility and antimicrobial activity, but also enables in situ monitoring of cumulative drug release, offering new insights and theoretical support for the design of wound adhesives for post-surgical internal sites.

## 4. Materials and Methods

### 4.1. Materials

All reagents and solvents were purchased directly and used as received, without any further purification. Neodymium(III) chloride hexahydrate, 2-amino-1,4-benzenedicarboxylic acid (H_2_ATA), LEV, sodium hyaluronate (HA) (98%), and nano-hydroxyapatite (HAp) were all purchased from McLean Limited; Type B gelatine was purchased from Baotou Dongbao Biotechnology Co., Ltd. (Shanghai, China); methacrylic anhydride was purchased from Aladdin Co., Ltd.; and lithium phenyl(2,4,6-trimethylbenzoyl) phosphate (LAP) was purchased from Adamas Reagents Co., Ltd. (Shanghai, China).

### 4.2. Synthesis Method

#### 4.2.1. Synthesis of Drug-Loaded Nd(ATA)

A total of 2 mmol of NdCl_3_·6H_2_O was added to 150 mL of distilled water to prepare an aqueous solution of NdCl_3_. A total of 2 mmol of H_2_ATA was added to 20 mL of ammonia solution, then titrated with hydrochloric acid to adjust the pH to 6. The H_2_ATA solution and the NdCl_3_ solution were mixed in a beaker and magnetically stirred in an oil bath at 50 °C for 30 min until a large amount of brown precipitate formed in the solution. The reaction mixture was left to stand at 60 °C for 2 h, after which the precipitate was separated by centrifugation. It was washed three times successively with deionised water and ethanol, then dried at 45 °C for 24 h to yield a brown powder of Nd(ATA). Nd(ATA) at a concentration of 2 mg/mL was soaked in a 5 × 10^−3^ M LEV solution for 24 h. The samples were treated with water under ultrasonic agitation for 3 min, then centrifuged until no drug peaks were detected in the supernatant upon UV analysis. Simultaneously, the supernatant was collected, and its absorbance was measured using a UV spectrophotometer to calculate the drug loading efficiency and evaluate the drug-loading capacity of Nd(ATA) for LEV (Equation (1)). The washed precipitate was dried in an oven at 45 °C for 24 h, ultimately yielding LEV@Nd(ATA).

(1) $$\mathrm{Drug}\;\mathrm{loading}\;\mathrm{capacity}\;\left(\%\right)=\frac{M_{0}-M_{1}}{M_{m}+(M_{0}-M_{1})}\;\times \;100\%$$

In Equation (1), M_0_, M_1_ and M_m_ represent the total amount of drug added, the amount of drug detected in the supernatant, and the amount of MOFs added, respectively.

#### 4.2.2. Synthesis of Composite Hydrogel

**Figure 9:**
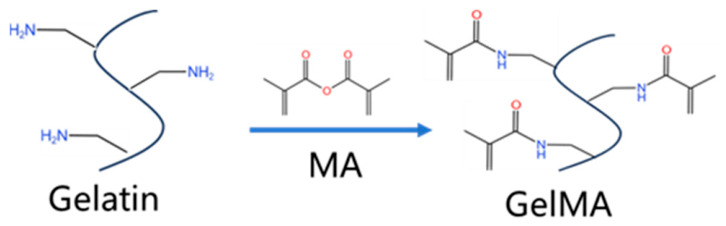
Schematic diagram of the GelMA reaction.

Dissolve 10 g of Type B gelatine in 100 mL of a Na_2_CO_3_-NaHCO_3_ buffer solution (pH 9–10) to prepare a gelatine solution. Slowly add 2.7 mL of methacrylic anhydride (MA) to the gelatine solution at 50 °C and stir under a magnetic stirrer for 3 h. Monitor the pH during the reaction and maintain a slightly alkaline environment. Upon completion of the reaction, add 200 mL of water, then ultrafiltrate until the conductivity is less than 10 μS/cm. After freeze-drying, a foamy 66% substituted methacryloylated gelatin (GelMA) is obtained. A schematic diagram of the reaction is shown in Figure 9.

Dissolve 1.2 g of GelMA in 10 mL of deionised water at 50 °C, then slowly add 0.2 g of HA, 0.5 g of HAp and 0.05 g of LAP in stages under magnetic stirring to prepare the gel precursor solution. Add 20 mg of LEV@Nd(ATA) to the gel precursor solution. Once the mixture has been stirred until homogeneous, pour it into a Petri dish and cross-link under a 365 nm UV lamp for 1–2 min to obtain the composite hydrogel LEV@Nd(ATA)-Gel.

### 4.3. Basic Characterization

The crystal structures and infrared spectra of Nd(ATA) and LEV@Nd(ATA) were determined using an X-ray diffractometer (Bruker AXS D2, Karlsruhe, Germany) and a Fourier transform infrared spectrometer (Nicolet, Madison, WI, USA), respectively. The morphology and specific surface area of Nd(ATA) were determined using a scanning electron microscope (SEM, Quattro S, Thermo Fisher, Waltham, MA, USA) and a pore size analyzer (Mike ASAP2460, Malvern, UK). The near-infrared emission of Nd(ATA) was measured using a steady-state/transient fluorescence spectrometer (FLS1000, Edinburgh, UK) under excitation at 808 nm. The nuclear magnetic resonance (NMR) spectra of Type B gelatin and GelMA were determined using a 600 MHz NMR spectrometer (AVANCE III HD 600 MHz, Bruker, Germany).

### 4.4. Mechanical Tests

**Figure 10:**
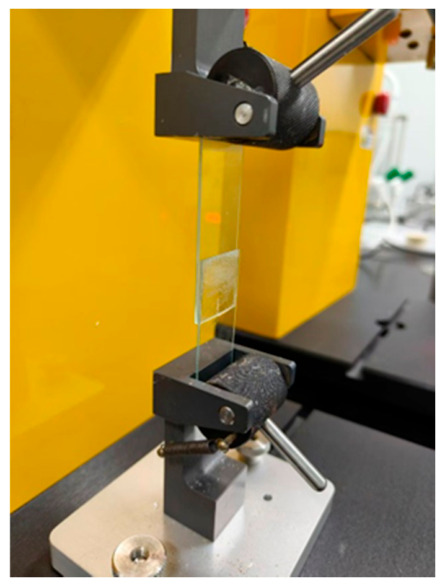
Schematic diagram of a texture analyzer in operation.

The tensile strength and adhesion strength of LEV@Nd(ATA)-Gel were measured using a texture analyzer (CT3, Brookfield, Middleboro, MA, USA) and the corresponding fixtures. A 20% gelatin solution (Type B) at 50 °C was applied to one end of a glass slide, forming a 2 cm × 2 cm area to simulate tissue conditions. After air-drying, 100 μL of the gel precursor solution was dispensed onto the coated area of one slide; the other slide was then pressed firmly against it and cross-linked under a UV lamp. The two slides were clamped between the texturiser’s fixtures (Figure 10), and the fixture movement speed was set to 0.5 mm/s until significant relative slippage occurred between the slides; the lap-shear strength was then recorded.

To determine the tensile strength of the composite hydrogel, cut the hydrogel into strips measuring 5 cm × 2 cm × 0.3 cm, clamp them between the two jaws of the texture analyzer, set the jaw movement speed to 0.5 mm/s, and continue until the hydrogel strip breaks completely; record the tensile strength value.

### 4.5. Biocompatibility Evaluation

#### 4.5.1. Swelling Ratio

The GelMa, Gel-HA, and LEV@Nd(ATA)-Gel samples were freeze-dried and weighed; the initial weight was recorded as M_0_. The gel samples were then immersed in PBS solution, removed after a specified time, and the surface moisture was wiped dry before measuring the wet weight M_t_ of the gels. The gel swelling ratio was calculated using the following Equation (2):

(2) $$Swelling\;ratio=\frac{M_{t}}{M_{0}}\times 100\%$$

#### 4.5.2. Degradation Rate

Hydrogels of different concentrations were freeze-dried and weighed; the initial weight was recorded as W_0_. The hydrogels were then immersed separately in PBS and in PBS containing 50 U/mL Type I collagenase. After a specified period, they were removed, freeze-dried, and weighed (W_t_). The degradation rate was calculated using the following Equation (3):

(3) $$Degradation\;rate=\frac{W_{0}-W_{t}}{W_{0}}\times 100\%$$

#### 4.5.3. Hemolysis Rate

The haemocompatibility of the composite hydrogel was assessed using New Zealand rabbit blood. Two milliliters of fresh anticoagulated whole blood from a New Zealand rabbit was diluted with 5 mL of saline solution, centrifuged at 3000 rpm for 10 min, and the supernatant was discarded. The resulting red blood cells were washed three times with saline solution, and the purified red blood cells were then further diluted to a final concentration of 2% (*v*/*v*) in suspension. A total of 10 mg of the composite hydrogel was placed in a centrifuge tube and 10 mL of saline solution was added; the positive control group used distilled water, and the negative control group used saline solution. The centrifuge tubes were incubated in a 37 °C water bath for 30 min. Subsequently, 200 μL of the red blood cell suspension (2% (*v*/*v*)) was added to each tube; after gentle mixing, the tubes were incubated in a 37 °C water bath for 1 h. Upon completion of the incubation, the tubes were centrifuged at 3000 rpm for 10 min, the supernatant was transferred to a new centrifuge tube for photography, and the absorbance of the supernatant was measured at a wavelength of 540 nm using a microplate reader. The haemolysis rate was calculated according to Equation (4).

#### 4.5.4. Cell Viability

The cytotoxicity of the composite hydrogel was assessed using L929 mouse fibroblasts, The L929 cell line was provided by Procell Life Science & Technology Co., Ltd. Samples were immersed in DMEM culture medium at an extraction concentration of 10 mg/mL for 24 h at 37 °C, with DMEM culture medium serving as the negative control. Five thousand L929 cells were seeded into each well of a 96-well plate, and 100 μL of DMEM cell culture medium supplemented with 10% fetal bovine serum (FBS) was added. The plates were cultured in a 37 °C constant-temperature incubator with 5% CO_2_. After 24 h, the medium was replaced with 100 μL of the gel extract and cultured for a further 24, 48 and 72 h. Upon completion of the culture, 10 μL of CCK-8 solution was added to each well and incubated for 1 h. Absorbance was then measured at 450 nm using a microplate reader (EPOCH 2, BioTek, Winooski, VT, USA), and cell viability was calculated using Equation (4). At the same time, L929 cells can be stained for live/dead cells using both calcein and propidium iodide solutions, and then observed under a fluorescence microscope [58].

(4) $$\mathrm{Relative}\;\mathrm{ratio}\;\left(\%\right)=\frac{A_{s}-A_{n}}{A_{p}-A_{n}}\times 100\%$$

In Equation (4), As, An and Ap represent the absorbance values of the supernatants from the experimental group, the negative control group and the positive control group, respectively.

#### 4.5.5. Cell Migration

Seed 8 × 10^5^ L929 cells into each well of a 6-well plate, add 2 mL of DMEM cell culture medium supplemented with 10% fetal bovine serum (FBS), and culture in a 37 °C incubator with 5% CO_2_ until the cell density reaches 90% or higher [58]. Use a sterile pipette tip to create a scratch. After washing away the scraped cells with sterile PBS, add 2 mL of gel extraction solution and continue culturing, using DMEM cell culture medium as the negative control. Take photographs at 0, 24 and 56 h for observation, and measure and analyze the scratch area using Image J software (version 1.54g, National Institutes of Health, Bethesda, MD, USA). Calculate the cell migration rate according to the following formula:

(5) $$Migration\;rate\left(\%\right)=\frac{A_{t}}{A_{0}}\times 100\%$$

In Equation (5), A_0_ and A_t_ represent the initial and t h scratch areas, respectively.

### 4.6. In Vitro Sustained-Release of LEV

LEV@Nd(ATA), LEV@Nd(ATA)-Gel, and LEV@Nd(ATA)-Gel that had been stored at 4 °C in the dark for five days were each dissolved at a concentration of 2 mg/mL in 20 mL of PBS solution (pH 3, 5, and 7). Supernatants were collected at specific time intervals and placed in cuvettes for UV-vis absorption spectroscopy (Cary-50UV-vis, Varian, Palo Alto, CA, USA). After each sample was taken, an equal volume of PBS solution of the same pH was replenished to the supernatant. Drug release experiments were conducted on LEV@Nd(ATA)-Gel samples before and after five days of storage at 4 °C. In PBS solution at pH 5; at 0, 3, 6, 12, 18, 24 h, the composite hydrogel samples were excited using an 808 nm laser, and their fluorescence images were captured using a CCD camera. Concurrently, a steady-state/transient fluorescence spectrometer was used to determine the fluorescence spectra before and after drug release in PBS solution at pH 5. Our experimental conditions ensure that the drug concentration in the aqueous solution remains below 10% to 20% of its solubility [59].

### 4.7. In Vitro Antimicrobial Properties Experiment

#### 4.7.1. In Vitro Antimicrobial Testing

Testing the Antimicrobial Activity of the Various Components in LEV@Nd(ATA)-Gel: Add 1,000 μL of 1 mg/mL solutions of the gel (excluding LEV@Nd(ATA)), Nd(ATA), LEV, and LEV@Nd(ATA)-Gel to separate centrifuge tubes, and sterilize them. Add 1,000 μL of a bacterial suspension at 1 × 10^8^ CFU/mL to the sterilized centrifuge tubes and incubate on a shaking incubator at 37 °C for 6 h. After incubation, dilute the mixture to 1×10^6^ CFU/mL. Take and evenly spread a 100 μL aliquot onto pre-prepared agar plates using a spreader rod. Incubate the plates at 37 °C for 18–24 h, and count the number of colonies to verify the antibacterial activity of the gel, Nd(ATA), LEV, and LEV@Nd(ATA)-gel.

Testing the Antibacterial Activity of LEV@Nd(ATA)-Gel at Different Volumes: Add 50, 75, 100, 300, 500, 700, and 1000 μL of LEV@Nd(ATA)-Gel to separate 1.5 mL centrifuge tubes. After solidification via UV irradiation, sterilize the tubes. Add 1 mL of a bacterial suspension at 1 × 10^8^ CFU/mL to each tube and incubate on a shaking incubator at 37 °C for 6 h. After incubation, dilute the mixture to 1 × 10^4^ CFU/mL. Then, evenly spread a 100 μL aliquot of the diluted solution onto pre-poured agar plates using a spreader, and incubate the plates at 37 °C for 18–24 h. The number of colonies were counted to verify the antimicrobial activity of the composite hydrogel upon drug release.

#### 4.7.2. Diameter of Inhibition Zone (DIZ) Assays

Take 100 μL of a bacterial suspension with a concentration of 1 × 10^8^ CFU/mL and spread it evenly over the surface of an agar plate; allow it to dry at room temperature for 5 min. Depending on the number of samples, place filter paper strips—which have been soaked in a sample solution with a concentration of 1 mg/mL—in different areas of the plate. Incubate the plate at 37 °C for 24 h, then measure the diameter of the inhibition zone (DIZ).

#### 4.7.3. Minimum Inhibitory Concentration (MIC) and Minimum Bactericidal Concentration (MBC)

Dilute the sample solution in a gradient (50, 100, 300, 500, 700, 1000, 2000, 3000 μg/mL) and add 100 μL of the diluted sample to each well of a 96-well plate. Next, add 100 μL of a bacterial suspension at 1 × 10^6^ CFU/mL to the corresponding wells. After incubating at 37 °C for 20 h, measure the absorbance of the samples at 600 nm. The MIC of a sample is the lowest concentration at which the absorbance no longer increases relative to the initial value. Take 100 μL of the mixture from the 96-well plate at which the absorbance no longer increases relative to the initial value, spread it on an agar plate, and incubate overnight; the lowest concentration at which no colonies grow is defined as the MBC level [60].

### 4.8. Statistical Analysis

Statistical analysis was performed using GraphPad Prism (version 10.1, GraphPad Software, San Diego, CA, USA). All quantitative data are presented as mean ± standard deviation (SD). Each experiment was independently repeated at least three times. Statistical comparisons between two groups were conducted using Student’s *t*-test. For comparisons among multiple groups, one-way analysis of variance (ANOVA) followed by Tukey’s post hoc test was applied. A value of *p* < 0.05 was considered statistically significant. Significant differences are indicated by asterisks (* *p* < 0.05, ** *p* < 0.01, *** *p* < 0.001, **** *p* < 0.0001), while groups with no statistically significant difference (*p* > 0.05) are not marked with symbols to maintain figure clarity.

## Data Availability

The original contributions of the study are included in the article. Further inquiries can be directed to the corresponding authors.
